# Reprogramming of the retinoic acid pathway in decidualizing human endometrial stromal cells

**DOI:** 10.1371/journal.pone.0173035

**Published:** 2017-03-02

**Authors:** Rie Ozaki, Keiji Kuroda, Yuko Ikemoto, Asako Ochiai, Akemi Matsumoto, Jun Kumakiri, Mari Kitade, Atsuo Itakura, Joanne Muter, Jan J Brosens, Satoru Takeda

**Affiliations:** 1 Department of Obstetrics and Gynaecology, Juntendo University Faculty of Medicine, Tokyo, Japan; 2 The Division of Biomedical Sciences, Clinical Science Research Laboratories, Warwick Medical School, Coventry, United Kingdom; 3 Tommy’s National Miscarriage Research Centre, University Hospitals Coventry & Warwickshire, Coventry, United Kingdom; Laboratoire de Biologie du Développement de Villefranche-sur-Mer, FRANCE

## Abstract

Upon breaching of the endometrial surface epithelium, the implanting embryo embeds in the decidualizing stroma. Retinoic acid (RA), a metabolite of vitamin A, is an important morphogen during embryonic and fetal development, although the role of the RA pathway in the surrounding decidual cells is not understood. Here we show that decidual transformation of human endometrial stromal cells (HESCs) results in profound reprogramming of the RA signaling and metabolism pathways. Differentiating HESCs downregulate the intracellular carrier proteins CRABP2 and FABP5, responsible for transfer and binding of RA to the nuclear receptors RAR and PPARβ/δ, respectively. Furthermore, the expression of RAR, the receptor that mediates the pro-apoptotic effects of RA, was also inhibited. By contrast, PPARβ/δ, which transduces the differentiation responses of RA, was upregulated. Decidualization was also associated with increased expression of retinol-binding protein 4 (RBP4) and various enzymes involved in the metabolism of RA and its precursor, retinaldehyde (Rald), including CYP26A1, DHRS3, and RDH12. Exposure of differentiating HESCs to RA or Rald reversed the inhibition of the CRABP2-RAR pathway, perturbed the expression of decidual marker genes and triggered cell death. Taken together, the data demonstrate that decidualizing HESCs silence RA signaling by downregulating key cytoplasmic binding proteins and by increasing retinoid metabolism. However, excessive RA exposure is toxic for decidual cells and triggers a response that may lead to pregnancy failure.

## Introduction

Decidualization denotes the transformation of endometrial stromal cells into specialized decidual cells that provide the nutritive and immune-privileged matrix for embryo implantation and placental formation [1]. Perturbed decidualization is associated with reproductive failure, including recurrent miscarriage [2–4]. Recently, several co-culture studies reported that decidualizing human endometrial stromal cells (HESCs) serve as biosensors of embryo quality at implantation [2, 5, 6]. In parallel with the typical morphological changes, differentiating HESCs express a number of decidual markers, including prolactin (PRL), insulin-like growth factor-binding protein-1 (IGFBP-1) and 11β-hydroxysteroid dehydrogenase type 1 (11βHSD1), the enzyme that converts inert cortisone into active cortisol [7–9]. Consequently, decidualization results in local cortisol production and activation of the glucocorticoid receptor (GR) and mineralocorticoid receptor (MR), which are both expressed in HESCs [7]. We have previously shown that MR targets and transcriptionally regulates several genes involved in retinoid metabolism in differentiating HESCs [7]. These observations indicated that decidualization may alter the retinoic acid (RA) pathway in HESCs.

Retinoids comprise a large family of versatile vitamin A (retinol) derivatives that regulate multiple cellular functions and confer immune tolerance [10, 11]. Retinoids, and more specifically the most active metabolite *all-trans* retinoic acid (atRA), are essential for visual function, adipocyte metabolism, reproduction and tissue homeostasis in various organs [12, 13]. In pregnancy, atRA acts as a critical morphogen for neural differentiation, body axis formation, and organogenesis in the developing embryo. Excessive levels are however toxic, necessitating tight regulation of transfer of maternal retinoids to fetus via the placenta [14, 15]. Retinol is generally acquired as provitamin A in the form of carotenoids, synthesized in leafy and yellow vegetables [16]. Most retinoids are stored as retinyl esters in inert retinoid containing lipid droplets in liver and adipose tissue [17, 18]. Retinol, hydrolyzed from retinyl ester, is transported to target tissues bound to retinol binding proteins (RBPs) or albumin. RA is then formed enzymatically from retinol by a two-step oxidation process in which the generation of retinaldehyde (Rald; also known as retinal) is followed by conversion of Rald in RA [10, 17, 19]. RA regulates gene expression by activating several members of the nuclear receptor family of transcription factors, including retinoic acid receptors (RARs) and peroxisome proliferator-activation receptor (PPAR) β/δ. Depending on preferential activation of PPAR β/δ or RAR, RA promotes cellular differentiation and survival or induces cell cycle arrest and apoptosis, respectively. This partitioning of the transcriptional RA response is tightly regulated by two intracellular lipid-binding proteins: cellular retinoic acid-binding protein 2 (CRABP2) and fatty acid-binding protein 5 (FABP5). Binding of RA to CRABP2 in the cytoplasm promotes its nuclear translocation and delivers RA to RAR, which leads to hetero-dimerization with retinoid X receptor (RXR) and activation of genes involved in cell cycle arrest and apoptosis. By contrast, FABP5 delivers RA to PPAR β/δ, which in turn promotes cellular differentiation [20–22]. Cytochrome P450 26A1 (CYP26A1), a protein encoded by *CYP26A1*, metabolizes RA, rendering it a key regulator of cellular RA levels. However, excess levels of Rald are also cytotoxic [23]. Rald is converted to retinol in the cytoplasm by members of the family of short-chain dehydrogenases/reductase (SDR), including dehydrogenases/reductase 3 (DHRS3, also known as retinal SDR1) and retinol dehydrogenase 12 (RDH12) [17, 24, 25], and then exported out of the cell by binding to retinol binding protein 4 (RBP4) [17, 26].

Evidence from several mammalian species, including humans, suggests that the RA pathway is highly regulated in the endometrium during the window of implantation and early pregnancy [7, 27, 28]. However, a detailed analysis of this pathway is as yet lacking. In this study, we have examined the expression of key components in RA signaling and metabolism in decidualizing HESCs, a model system that closely recapitulates the *in vivo* changes in the stromal compartment during luteal phase of the cycle.

## Materials and methods

### Patient selection and endometrial biopsy

This study was approved by the local ethical committee of Juntendo University, Faculty of Medicine (No.14-103). Endometrial tissue samples were obtained following informed written consent. Biopsies were obtained from premenopausal women without overt uterine pathology and not receiving hormonal therapy or infertility treatment in the Department of Obstetrics and Gynecology of Juntendo University Hospital (Tokyo, Japan). Endometrial biopsies were timed between 7 to 11 days after the pre-ovulatory luteinizing hormone surge by using an endometrial suction curette TM (MX140, Fuji Medical, Tokyo, Japan). The biopsies were processed for primary cell culture.

### Primary human endometrial cell culture

Endometrial tissues were collected in Dulbecco’s modified Eagle’s medium (DMEM; 085456, Nacalai Tesque, Kyoto, Japan) containing 1% antibiotic solution. The tissue were finely minced and enzymatically digested with 5 mg collagenase (5 mg / 100 μl) (C9891, SIGMA, Saint Louis, USA) and deoxyribonuclease (DNase) type I (100 μg / μl) (11284932001, Roche Applied Science, Mannheim, Germany) for 1h at 37°C. After centrifugation, the pellet was suspended in cultured medium of DMEM containing 10% dextran-coated charcoal (DCC) treated fetal bovine serum (FBS; 172012, SIGMA, Saint Louis, USA), 1% antibiotic solution, 1% L-glutamine. HESCs were cultured until confluence in 75 cm^2^ culture flasks and passaged once or twice. For decidualizing HESCs, confluent monolayers were maintained in DMEM without phenol red (REF11039-021, GIBCO, life technologies, Grand Island, USA) containing 2% DCC-FBS and treated with 0.5 mM 8-bromoadenosine 3’5’-cyclic AMP (8-bromo-cAMP; B7880,SIGMA,USA), 1 μM progesterone (P_4_; P0130, SIGMA, China), and 0.1 μM cortisone (E; C2755,SIGMA,China) in combination with 10^−8^ M to 10^−11^ M *all trans*-RA (atRA; R2625, SIGMA, China) or 10^−8^ M *all-trans* Rald (atRald; R2500,SIGMA,China). Normal serum levels of vitamin A are between 1–2.5 × 10^−6^ M [29]. A previous study showed that atRA at a concentration of 10^−8^ M or higher inhibits decidualization of HESCs [30]. Therefore, we use atRA at 10^−8^ to 10^−11^ M and atRald at 10^−8^ M in the current study. The compounds and treated samples were protected from light in line with the manufacturer’s instructions. Inactive cortisone (E) was added to decidualizing cell cultures as the substrate for endogenous conversion to active cortisol by 11βHSD1 [7].

### Real-Time Quantitative PCR (RTQ-PCR)

Total RNA was extracted from primary cell cultures with RNeasy plus mini kit (741134, QIAGEN, Hilden, Germany). And complementary DNA was generated using the SuperScript II Reverse Transcriptase for RT-PCR kit (18064, Invitrogen Ltd. Life technology, California, USA). Template quantification was performed with 7500 fast real-time PCR system (4351106, Applied Biosystems, Forster City, USA) using power fast SYBR Green Master Mix (4385612, Applied Biosystems, Forster City, USA) as dye layer and the relative standard curve calculation method. RNA input variances were normalized against the levels of *L19* housekeeping gene, which encodes a ribosomal protein. All measurements were performed in duplicate. Specific primer pairs were designed using primer 3 software (http://frodo.wi.mit.edu): L19 sense,5’- GCG GAA GGG TAC AGC CAA T-3’, L19-R antisense, 5’- GCA GCC GGC GCA AA-3’; decidual PRL sense 5’-AAG CTG TAG AGA TTG AGG AGC AAA C-3’ and decidual PRL antisense, 5’-TCA GGA TGA ACC TGG CTG ACT A-3’; IGFBP1 sense, 5’-CGA AGG CTC TCC ATG TCA CCA-3’ and IGFBP1 antisense, 5’-TGT CTC CTG TGC CTT GGC TAA AC-3’; 11βHSD1 sense,5’-AGC AAG TTT GCT TTG GAT GG-3’ and 11βHSD1 antisense, 5’-AGA GCT CCC CCT TTG ATG AT-3’; CRABP2 sense, 5’-TGT GAG CAG AAG CTC CTG AAG-3’ and CRABP2 antisense 5’-GTT CTA CCT GTG GCC ACT CAC T-3’; RARα sense, 5’- GCC CAG CTC ACC ACA TCT TC-3’ and RARα antisense, 5’- GGA GCA ATG GCT TGT GAG TTC T-3’; RXRα sense, 5’- GTC CTT GGA GGC CTA CTG CAA-3’ and RXRα antisense, 5’- CCG ATG AGC TTG AAG AAG AAG AGA T-3’; PPARβ/δ sense, 5’- ACT GAC CCA ACT GAT CCT GCT C-3’ and PPARβ/δ antisense,5’- GCC TGG CAA ACC AGT GTG AA-3’; DHRS3 sense, 5’-GAA GCT GTG CAG CTC AAC CA-3’ and DHRS3 antisense, 5’-CAT GCA GGT GTA GGT TCC TGA GA-3’; RDH12 sense, 5’- GGG AGT CTG CTG CCA GTG AA-3’ and RDH12 antisense, 5’- CCA TCA GCT GTC TTG GAA TAT GGA-3’; RBP4 sense, 5’- AGG CTC GCT TCT CTG GGA CC-3’ and RBP4 antisense, 5’- CCC TTG GCT GTG GCG CTC AT-3’. We used commercially available primers for FABP5 (330001 PPH02412E, QIAGEN, Mayland, USA) and Cyp26a1 (330001 PPH01229A, QIAGEN, Mayland, USA).

### Western blot analysis

Whole-cell protein extracts were obtained by direct lysis in SDS sample buffer heated to 75°C. Proteins resolved by SDS-PAGE were transferred to a polyacrylamide membrane (Bio-rad laboratories, Calfornia, USA.) and probed with antibodies raised against 11βHSD1, 1:5000 (AB83552, Abcam, UK); CRABP2, 1:5000 (HPA004135, SIGMA, Stockholm, Sweden); FABP5, 1:1000 (AB37267, Abcam, UK); RARα, 1:1700, (AB28767, Abcam, UK); RXRα,1:1000 (5388S, Cell Signaling, Danvers, USA); PPARβ/δ, 1:1000 (AB8937, Abcam, UK); Cyp26a1, 1:2000 (SC53618, SantaCruz); DHRS3, 1:200 (15393-1-AP; Proteintech Group Manchester, UK); RBP4,1:1000 (SC46688, SantaCruz); RDH12, 1:1000 (AB87038, Abcam, UK); and β-actin,1:5000 (A2066, SIGMA, UK). After incubation with peroxidase-conjugated secondary antibody, anti-rabbit or anti-mouse IgG diluted 1:1000–1:50,000 (Jackson Immunoresearch Laboratories, West Grobe, USA), detection of chemiluminescence reaction was visualized using the ECL plus chemiluminescence kit (PCC-NCI 32132 Amersham, GE Healthcare, Japan) and normalized to β-actin. The Western blots were quantified using ImageJ software.

### Cell proliferation and viability assay

Real-time adherent cell proliferation in HESCs were determined by the label-free xCELLigence Real-Time Cell Analyzer (RTCA) DP system (Roche Diagnostics, Basel, Switzerland) as described in previous literature [31]. Briefly, HESCs were cultured in 10% DCC-FBS until 80% confluent into 16-well plates, containing an interdigitized gold microelectrode (E-plate-16; Roche Diagnostics, Basel, Switzerland) on which cells attach and proliferate. Cell contact acts to increase impedance across the gold electrodes and is reported as an arbitrary “cell index” value, which is an indication of confluency and adherence. The RTCA DP instrument was placed at 37°C in a humidified environment with 95% air and 5% CO_2_ and individual wells within the E-plate-16 were monitored first every 15 min for 3 h and then hourly for 60 h. Changes in cell index were captured and analyzed using the RTCA Software v1.2. Cellular viability was determined by the cell’s ability to reduce tetrazolium salts (XTT) into colored formazan compounds by mitochondrial enzymes. XTT detection solution (50 μl) was added to 100 μl of cultured cells in a 96 well format. The plate was incubated at 37°C for 4 hours. Dye absorbance was measured at 450 nm on the PHERAStar FS microplate reader (BMG Labtech, Offenburg, Germany).

### Statistical analysis

Data presented in this study are representative of four or more biological replicates. Statistical analysis was performed using one-way analysis of variance (ANOVA) and Student’s t test or Mann-Whitney *U* test after normalization of the data with SPSS software. The level of significance was defined as *P* < 0.05.

## Results

### Expression of intracellular RA binding proteins and receptors in undifferentiated and decidualizing HESCs

**Fig 1:**
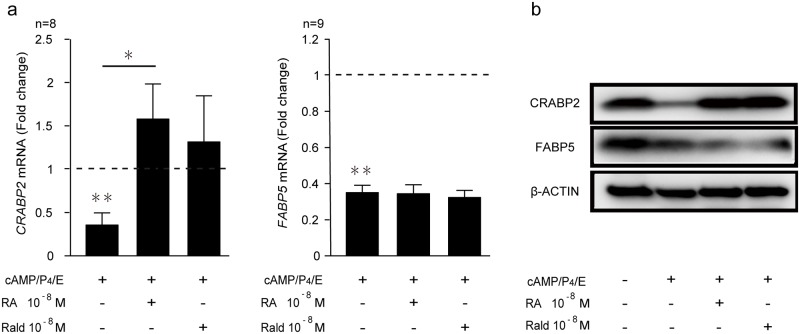
Expression of cellular retinoic acid binding proteins in decidualizing HESCs. **(a)** *CRABP2* and *FABP5* transcript levels in undifferentiated or decidualized cells treated with 8-bromo-cAMP, P4, and E in combination with or without 10^−8^ M atRA or 10^−8^ M atRald for 8 days. The results show fold-change (mean ± SEM) relative to vehicle control (*dotted lines*). *CRABP2* mRNA was measured in 8 primary HESC cultures and *FABP5* mRNA in 9 cultures. **(b)** Representative Western blot analysis of retinoic acid binding proteins in whole cell lysates from undifferentiated or cells decidualized in the absence or presence of 10^−8^ M atRA or 10^−8^ M atRald. β-actin served as a loading control. E, cortisone; RA, retinoic acid; Rald, retinaldehyde. * indicates *P* < 0.05; ** *P* < 0.01.

**Fig 2:**
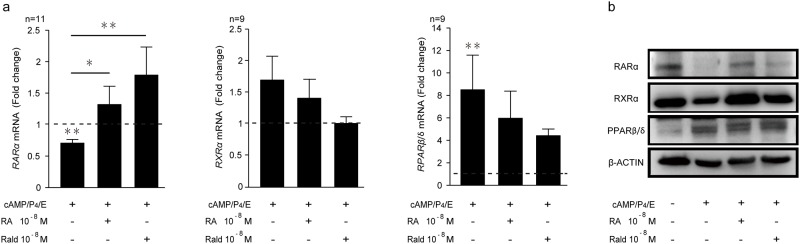
Expression of RA nuclear receptors in decidualizing HESCs. **(a)** Transcript levels of *RARα*, *RXRα* and *PPARβ/δ* in undifferentiated or cells in the presence or absence of 10^−8^ M atRA or 10^−8^ M atRald for 8 days (n = 9–11). Transcripts levels were normalized to expression in vehicle-treated control cells (*dotted line*). **(b)** Representative Western blot analysis of a parallel culture. β-actin served as a loading control. * *P* < 0.05; ** *P* < 0.01.

Cellular sensitivity to RA is primarily determined by its subnanomolar affinity for the intracellular binding proteins CRABP2 and FABP5, which deliver RA directly to its cognate nuclear receptors [20–22]. The expression of these binding proteins was examined in undifferentiated primary HESCs and cells decidualized for 8 days in the presence or absence of atRA or atRald. Parallel cultures were harvested for CRABP2 and FABP5 expression levels measured by RTQ-PCR and Western blot analysis. As shown in Fig 1a, *CRABP2* and *FABP5* transcript levels declined by 63% and 65% in the cells decidualized for 8 days, respectively. In fact, down-regulation of these binding proteins at mRNA level was also already apparent after 4 days of decidualization and maintained over a 12-day time-course (S1 Fig). Western blot analysis in undifferentiated HESCs and cells decidualized for 8 days revealed that loss of CRABP2 protein was more pronounced when compared to FABP5. Interestingly, co-treatment of decidualizing cells with atRA or atRald completely reversed the down-regulation of CRABP2 but not FABP5 (Figs 1a and 2a). The data show that the relative expression of these critical intracellular lipid-binding proteins changes upon decidualization in favor of FABP5. However, in the presence of atRA or atRald, the balance is reversed, rendering CRABP2 the dominant cytoplasmic RA binding protein.

The RA-responsive nuclear receptors, RAR and PPARβ/δ, form heterodimers with RXR, leading to activation of distinct transcriptional networks. Upon decidualization, RARα declined significantly at both mRNA (Fig 2a and S1 Fig) and protein level (Fig 2b and S2 Fig). In parallel, we observed a reduction in RXRα protein but not mRNA level (Fig 2). PPARβ/δ levels increased markedly upon decidualization. Co-treatment of decidualizing cultures with atRA and atRald, reversed the decrease in RARα expression whereas the induction of PPARβ/δ was unaffected (Fig 2a and S2 Fig).

Taken together, the relative loss of CRABP2 and FABP5 expression upon decidualization of HESCs suggests that the cells become relatively resistant to atRA signaling. In addition, the predominance of PPARβ/δ in differentiating HESCs suggests a potential role for this signaling pathway in the regulation of decidual genes. However, exposure of decidualizing cells to atRA or atRald completely reverses these changes and establishes a dominance of the CRABP2-RAR pathway.

### Induction of retinoid metabolizing genes in decidualized HESCs

**Fig 3:**
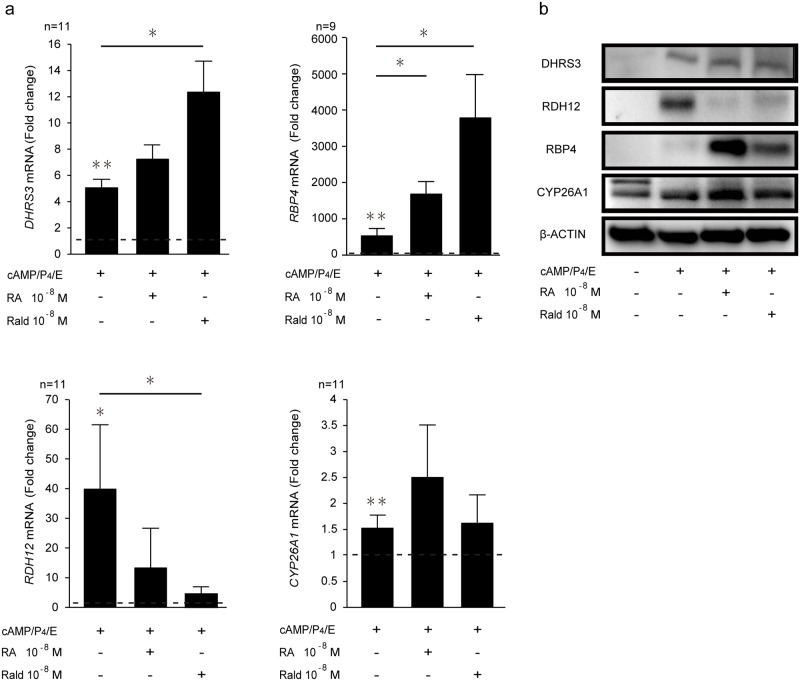
Induction of RA metabolic enzymes in decidualizing cells. **(a)** Expression of *DHRS3*, *RDH12*, *RBP4* and *CYP26A1* transcripts in undifferentiated or cells decidualized with 8-bromo-cAMP, P4, and E in the presence or absence of 10^−8^ M atRA or 10^−8^ M atRald for 8 days (n = 9–11). The results show fold-change (mean ± SEM) in mRNA levels relative to expression levels in vehicle-treated undifferentiated HESCs (*dotted lines*). **(b)** Representative Western blot analysis of culture treated in parallel. β-actin served as a loading control. * *P* < 0.05; ** *P* < 0.01.

DHRS3 acts as a retinal reductase that converts Rald into retinol [17, 24, 25]. Decidualization was associated with a marked increase in DHRS3 expression, both at mRNA and protein level (Fig 3 and S2 Fig). Exogenous atRald but not atRA significantly enhanced the induction, at least at transcript levels. Decidualization was also associated with a strong upregulation of RDH12, although the induction was blunted when the cultures were exposed to atRA and atRald (Fig 3 and S2 Fig). The retinol binding protein RBP4 and the RA metabolizing enzyme CYP26A1 were upregulated upon decidualization. Co-treatment of the decidualizing cultures with atRA and atRald markedly enhanced the induction of RBP4 but not CYP26A1 (Fig 3 and S2 Fig).

### RA inhibits decidualization

**Fig 4:**
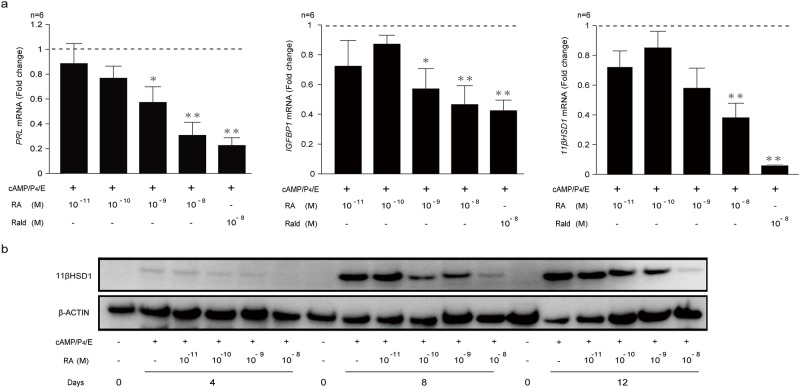
Impaired decidualization in responses to exogenous atRA and atRald. **(a)** RTQ-PCR analysis of *PRL*, *IGFBP1* and *HSD11B1* transcript levels in decidualizing HESCs treated with or without 10^−8^–10^−11^ M atRA or 10^−8^ M atRald for 8 days in 6 primary HESC cultures. The results show fold-change (mean ± SEM) relative to transcript levels in vehicle-treated control cells (*dotted lines*). **(b)** Western blot analysis of 11βHSD1 expression in protein lysates from primary undifferentiated cells and cultures decidualized for 4, 8 or 12 days in the presence of increasing concentrations of atRA or 10^-8^M at Rald. β-actin served as a loading control.

The profiling of key components in RA signaling pathway and retinoid metabolism indicate that decidualizing cells actively limit RA signaling by down-regulating cytoplasmic binding proteins and by increasing metabolism of RA and its precursor Rald. We also demonstrated that this reprogramming of the RA pathway is reversed upon co-treatment with atRA or atRald. To test the consequences of RA signaling in differentiating HESCs, primary cultures were decidualized in the presence of increasing concentrations of atRA, ranging from 10^−11^ to 10^−8^ M. In addition, some cultures were treated with atRald (10^−8^ M). As shown in Fig 4a, atRA inhibited the expression of key decidual marker genes, *PRL*, *IGFBP1* and *HSD11B1* (coding 11βHSD1), in a dose-dependent manner. However, the reduction in *PRL* and *IGFBP1* transcripts was only significant upon treatment with 10^−9^ M RA whereas inhibition of *HSD11B1* required 10^−8^ M RA. At 10^−8^ M, atRald was as effective as atRA in inhibiting the expression of decidual markers. To validate these observations, total protein lysates of cells decidualized for 4, 8 or 12 days in the presence of increasing concentrations of atRA were subjected to Western blot analysis and immunoprobed for 11βHSD1 expression. As shown in Fig 4b, a dose-dependent reduction in 11βHSD1 expression was observed at every time-point. Notably, 11βHSD1 repression seemed more effective upon treatment with 10^−8^ M atRald than with atRA at the same concentration (Fig 4).

**Fig 5:**
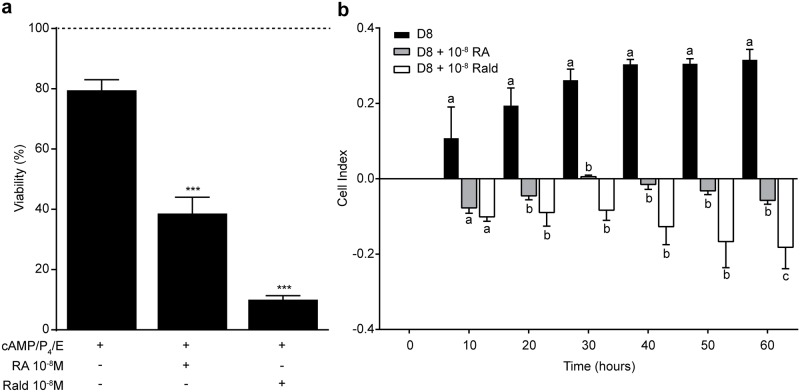
Cytoxicity of atRA and atRald in decidualizing HESCs. **(a)** Cell viability of primary HESCs, decidualized in the presence or absence of 10^−8^ M atRA or 10^−8^ M atRald, was analyzed by XTT assay. The data show % of viable cells (± SEM) relative to matched undifferentiated HESCs (*dotted line*) in 3 independent primary cultures. * *P* < 0.05; ** *P* < 0.01. **(b)** Parallel cultures were analysed over 60 hours using xCELLigence Real-Time Cell Analyzer (RTCA). Cell contact is monitored as an arbitrary “cell index” value, which is an indication of confluency and adherence. Different letters above the error bars indicate that those groups are significantly different from each other at *P* < 0.05. The results show mean ± SEM of 3 biological repeat experiments.

Because exposure to retinoids upregulates the CRABP2-RAR pathway in decidualizing cells, we postulated that treatment of decidual cells with either atRA or atRald would trigger cell death and block expansion of decidual cultures. To test this hypothesis, cellular viability and real-time cell proliferation were measured by an XTT assay and a label free xCELLigence Real-Time Cell Analyzer (RTCA), respectively. Cellular viability was reduced by 42% in the presence of 10^−8^ M atRA and 80% in the presence of atRald (Fig 5a). Furthermore, treatment with either atRA or atRald negatively impacted on the expansion of decidualizing HESCs (Fig 5b). The detrimental effect of atRald on HESCs proliferation was more pronounced when compared to atRA after 60 hours of monitoring (Fig 5b).

## Discussion

**Fig 6:**
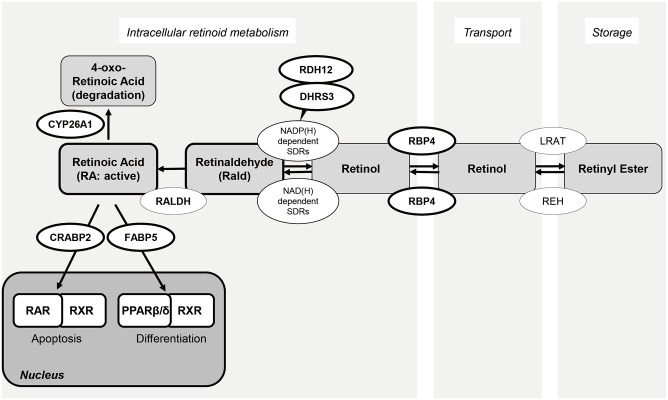
Schematic overview of the RA pathway. Retinol, hydrolyzed from retinyl ester, is converted to retinaldehyde (Rald) and generates retinoic acid (RA) following two-step oxidation. Intracellular RA binds to CRABP2 and activates heterodimer, RAR and RXR, leading to apoptotic machinery. By contrast, RA-dependent activation of nuclear receptor, PPAR β/δ is mediated by FABP5.

Decidual cells play a crucial role in ensuring survival of an implanting embryo by providing protection against a variety of environmental stressors and cytotoxic agents [1]. For the developing embryo and fetus, retinoids are essential but also potentially teratogenic. Here we report that the RA pathway, as depicted in Fig 6, is profoundly altered upon decidualization, exemplified by: (i) decreased expressions of RA binding protein, CRABP2 and FABP5; (ii) down-regulation of RARα but induction of PPARβ/δ; (iii) strong induction of DHRS3 and RDH12, enzymes involved in conversion of Rald to retinol; and (iv) induction of the catabolizing enzyme CYP26A1 and up-regulation of the retinol transporter RBP4. Taken together, the data suggest that decidualization attenuates cellular RA signaling and rebalances the downstream RA-responsive nuclear receptors in favor of PPARβ/δ. In the rat uterus, PPARβ/δ is highly expressed at implantation sites [27], suggesting a role RA-dependent PPARβ/δ activation during decidualization. However, we found no evidence that atRA, even at very low concentrations, enhances HESC differentiation, at least not when assessed by the induction of highly responsive decidual marker genes (*PRL*, *IGFBP1* and *HSD11B1*). At higher concentrations, atRA, as well as atRald, re-establishes the dominance of the CRABP2-RARα pathway in decidualizing cells, which in turn inhibited the expression of decidual markers and compromised cell viability. This observation is agreement with another study demonstrating that CRABP2 knockdown increases survival of HESCs [32]. Further, the lower ratio of CRABP2:FABP5 in ectopic endometrial cells has been implicated in the resistance of endometriotic lesions to apoptosis [32].

DHRS3 and RDH12 are members of the SDR family and convert Rald to retinol. Both enzymes are induced upon decidualization. However, exposure of decidualizing HESCs to atRA or atRald attenuated the upregulation of RDH12 but had no effect on the induction of DHRS3, at least not at protein level. RDH12 protects against atRald toxicity through its oxidation to all-trans retinol [33, 34]. However, RDH12 can simultaneously catalyze 11-cis retinol to 11-cis Rald [25]. DHRS3, which is also known as lipid droplet protein, induces storage of retinoids as cellular retinyl esters, contributing to protection from local RA or Rald toxicity [24, 35, 36]. DHRS3 has been shown to play and important role in suppression of RA signaling in embryos [36]. Furthermore, RBP4, which is highly upregulated in decidualizing cells exposed to atRA or atRald, binds not only retinol but also Rald directly [23]. Together with the induction of CYP26A1, a major RA-catabolizing enzyme, these observations indicate that decidualizing HESCs employ several mechanisms to control precisely cellular concentrations of bioactive retinoids.

Several studies have highlighted the importance of the RA pathway at implantation. For example, flushing of the mouse uterus with conditioned medium of human pre-implantation IVF embryos that subsequently implanted successfully resulted in strong induction of *Cyp26a1* and *Rdh12* and marked down-regulation of *Crabp2* [6]. In contrast to successful embryos, conditioned medium from developmentally impaired human embryos failed to modulate the expression of these genes in the mouse uterus [6]. Another study demonstrated that uterine injection of cyp26a1-specific antisense oligos or anti-cyp26a1 antibody on day 3 of pregnancy blocks implantation in mice [37]. Furthermore, several genes encoding intermediates in the RA pathway, including *CRABP2*, *DHRS3*, *RARRES1* and *RARRES3*, feature on the endometrial receptivity array (ERA), which was designed to discriminate between a receptive and non-receptive endometrium in women undergoing IVF treatment [38]. Finally, recurrent miscarriage has been linked to aberrant endometrial expression of CRABP2 during the window of receptivity [39]. Taken together, these reports indicate that the changes in primary decidualizing HESCs reflect the reprogramming of the RA pathway in the endometrium at implantation. However, additional studies are needed to examine the regulation of this pathway in endometrial epithelial cells.

## Conclusion

Decidual transformation of the endometrium is associated with wholesale reprogramming of both retinoid signaling and metabolism pathways. This reprogramming suggests resistance to RA and Rald signaling. However, decidual cells remain exquisitely sensitive to exogenous RA and Rald and mount a response that may increase the risk of early pregnancy failure but also decreases the likelihood of congenital abnormalities.

## Supporting information

S1 Fig Time-course analysis of decidualizing HESCs. RTQ-PCR analysis of *CRABP2*, *FABP5*, *RARa* and *PPAR β/δ* transcript levels in decidualizing HESCs for 4, 8 and 12 days. The results show fold-change (mean ± SEM) relative to transcript levels in vehicle-treated control cells (*dotted lines*). Different letters above the error bars indicate that those groups are significantly different from each other at *P* < 0.05. The results show mean ± SEM of 7–11 different primary cultures.
(TIF)

S2 Fig Quantification of protein expression. Western blots were quantified by densitometry using ImageJ software. The abundance of the indicated proteins was normalized to β-actin. The data show relative change in protein expression upon decidualization in the presence or absence atRA or Rald exposure was determined. The number of biological repeat experiments are indicated.
(TIF)

## References

[1] Gellersen B, Brosens JJ. Cyclic Decidualization of the human endometrium in reproductive health and failure. Endocr Rev. 2014;35(6):851–905. 10.1210/er.2014-1045 25141152

[2] Salker M, Teklenburg G, Molokhia M, Lavery S, Trew G, Aojanepong T, et al Natural selection of human embryos: impaired decidualization of endometrium disables embryo-maternal interactions and causes recurrent pregnancy loss. PLoS One. 2010;5(4):e 10287. 10.1371/journal.pone.0010287 PMC2858209 20422017

[3] Salker MS, Christian M, Steel JH, Nautiyal J, Lavery S, Trew G, et al Deregulation of the serum- and glucocorticoid-inducible kinase SGK1 in the endometrium causes reproductive failure. Nat Med. 2011;17(11):1509–13. 10.1038/nm.2498 22001908

[4] Weimar CHE, Kavelaars A, Brosens JJ, Gellersen B, de Vreeden-Elbertse JMT, Heijnen CJ, et al Endometrial stromal cells of women with recurrent miscarriage fail to discriminate between high-and low-quality human embryos. PLoS One. 2012;7(7). 10.1371/journal.pone.0041424 PMC3405140 22848492

[5] Teklenburg G, Salker M, Molokhia M, Lavery S, Trew G, Aojanepong T, et al Natural selection of human embryos: decidualizing endometrial stromal cells serve as sensors of embryo quality upon implantation. PLoS One. 2010;5(4). 10.1371/journal.pone.0010258 PMC2858159 20422011

[6] Brosens JJ, Salker MS, Teklenburg G, Nautiyal J, Salter S, Lucas ES, et al Uterine selection of human embryos at implantation. Sci Rep. 2014;4. 10.1038/srep03894 PMC3915549 24503642

[7] Kuroda K, Venkatakrishnan R, Salker MS, Lucas ES, Shaheen F, Kuroda M, et al Induction of 11 beta-HSD 1 and activation of distinct mineralocorticoid receptor- and glucocorticoid receptor-dependent gene networks in decidualizing human endometrial stromal cells. Mol Endocrinol. 2013;27(2):192–202. 10.1210/me.2012-1247 23275455 PMC5417328

[8] Courtney R, Stewart PM, Toh M, Ndongo M-N, Calle RA, Hirshberg B. Modulation of 11 beta-hydroxysteroid dehydrogenase (11 beta HSD) activity biomarkers and pharmacokinetics of PF-00915275, a selective 11 beta HSD1 inhibitor. J Clin Endocrinol Metab. 2008;93(2):550–6. 10.1210/jc.2007-1912 17986636

[9] Brosens JJ, Hayashi N, White JO. Progesterone receptor regulates decidual prolactin expression in differentiating human endometrial stromal cells. Endocrinol. 1999;140(10):4809–20. 10.1210/endo.140.10.7070 10499541

[10] Napoli JL. Retinoic acid biosynthesis and metabolism. FASEB J. 1996;10(9):993–1001. 8801182 10.1096/fasebj.10.9.8801182

[11] Napoli JL. Biosynthesis and metabolism of retinoic acid: roles of CRBP and CRABP in retinoic acid: rolesof CRBP and CRABP in retinoic acid homeostasis. J Nutr. 1993;123(2):362–6. 8381481 10.1093/jn/123.suppl_2.362

[12] Mark M, Ghyselinck NB, Chambon P. Function of retinoid nuclear receptors. Lessons from genetic and pharmacological dissections of the retinoic acid signaling pathway during mouse embryogenesis. Annu Rev Pharmacol Toxicol. 2006;46:451–80. 10.1146/annurev.pharmtox.46.120604.141156 16402912

[13] Clagett-Dame M, DeLuca HF. The role of vitamin A in mammalian reproduction and embryonic development. Annu Rev Nutr. 2002;22:347–81. 10.1146/annurev.nutr.22.010402.102745E 12055350

[14] Geelen JA. Hypervitaminosis A induced teratogenesis. CRC crit rev toxicol. 1979;6(4):351–75. 389569 10.3109/10408447909043651

[15] Collins MD, Mao GE. Teratology of retinoids. Annu Rev Pharmacol Toxicol. 1999;39:399–430. 10.1146/annurev.pharmtox.39.1.399 10331090

[16] Pilch SM. Analysis of vitamin A data from the health and nutrition examination surveys. J Nutr. 1987;117(4):636–40. 3585513 10.1093/jn/117.4.636

[17] Kedishvili NY. Enzymology of retinoic acid biosynthesis and degradation. J Lipid Res. 2013;54(7):1744–60. 10.1194/jlr.R037028 23630397 PMC3679379

[18] Blaner WS, O'Byrne SM, Wongsiriroj N, Kluwe J, D'Ambrosio DM, Jiang HF, et al Hepatic stellate cell lipid droplets: A specialized lipid droplet for retinoid storage. Biochim Biophys Acta. 2009;1791(6):467–73. 10.1016/j.bbalip.2008.11.001 19071229 PMC2719539

[19] Duester G, Mic FA, Molotkov A. Cytosolic retinoid dehydrogenases govern ubiquitous metabolism of retinol to retinaldehyde followed by tissue-specific metabolism to retinoic acid. Chem Biol Interact. 2003;143:201–10. 12604205 10.1016/s0009-2797(02)00204-1

[20] Wolf G. Retinoic acid as cause of cell proliferation or cell growth inhibition depending on activation of one of two different nuclear receptors. Nutr Rev. 2008;66(1):55–9. 10.1111/j.1753-4887.2007.00006.x 18254885

[21] Schug TT, Berry DC, Shaw NS, Travis SN, Noy N. Opposing effects of retinoic acid on cell growth result from alternate activation of two different nuclear receptors. Cell. 2007;129(4):723–33. 10.1016/j.cell.2007.02.050 17512406 PMC1948722

[22] Shaw N, Elholm M, Noy N. Retinoic acid is a high affinity selective ligand for the peroxisome proliferator-activated receptor beta/delta. J Biol Chem. 2003;278(43):41589–92. 10.1074/jbc.C300368200 12963727

[23] Ziouzenkova O, Orasanu G, Sharlach M, Akiyama TE, Berger JP, Viereck J, et al Retinaldehyde represses adipogenesis and diet-induced obesity. Nat Med. 2007;13(6):695–702. 10.1038/nm1587 17529981 PMC2233696

[24] Deisenroth C, Itahana Y, Tollini L, Jin AW, Zhang YP. p53-inducible DHRS3 Is an Endoplasmic Reticulum Protein Associated with Lipid Droplet Accumulation. J Biol Chem. 2011;286(32):28343–56. 10.1074/jbc.M111.254227 21659514 PMC3151078

[25] Haeseleer F, Jang GF, Imanishi Y, Driessen C, Matsumura M, Nelson PS, et al Dual-substrate specificity short chain retinol dehydrogenases from the vertebrate retina. J Biol Chem. 2002;277(47):45537–46. 10.1074/jbc.M208882200 12226107 PMC1435693

[26] D'Ambrosio DN, Clugston RD, Blaner WS. Vitamin A Metabolism: An Update. Nutr. 2011;3(1):63–103. 10.3390/nu3010063 PMC3042718 21350678

[27] Ding NZ, Ma XH, Diao HL, Xu LB, Yang ZM. Differential expression of peroxisome proliferator-activated receptor delta at implantation sites and in decidual cells of rat uterus. Reprod. 2003;125(6):817–25. 10.1530/rep.0.1250817 12773104

[28] Zheng WL, Sierra-Rivera E, Luan J, Osteen KG, Ong DE. Retinoic acid synthesis and expression of cellular retinol-binding protein and cellular retinoic acid-binding protein type II are concurrent with decidualization of rat uterine stromal cells. Endocrinol. 2000;141(2):802–8. 10.1210/endo.141.2.7323 10650963

[29] Safavi K. Serum vitamin A levels in psoriasis: results from the First National Health and Nutrition Examination Survey. Arch Dermatol. 1992;128(8):1130–1. 1497375

[30] Brar AK, Kessler CA, Meyer AJ, Cedars MI, Jikihara H. Retinoic acid suppresses in-vitro decidualization of human endometrial stromal cells. Mol Hum Reprod. 1996;2(3):185–93. 9238678 10.1093/molehr/2.3.185

[31] Muter J, Lucas ES, Chan Y-W, Brighton PJ, Moore JD, Lacey L, et al The clock protein period 2 synchronizes mitotic expansion and decidual transformation of human endometrial stromal cells. FASEB J. 2015;29(4):1603–14. 10.1096/fj.14-267195 25573754 PMC4396614

[32] Pavone ME, Reierstad S, Sun H, Milad M, Bulun SE, Cheng Y-H. Altered retinoid uptake and action contributes to cell survival in endometriosis. J Clin Endocrinol Metabol. 2010;95(11):E300–E9. 10.1210/jc.2010-0459 PMC2968735 20702525

[33] Maeda A, Maeda T, Imanishi Y, Sun W, Jastrzebska B, Hatala DA, et al Retinol dehydrogenase (RDH12) protects photoreceptors from light-induced degeneration in mice. J Biol Chem. 2006;281(49):37697–704. 10.1074/jbc.M608375200 17032653 PMC4124513

[34] Chen Y, Okano K, Maeda T, Chauhan V, Golczak M, Maeda A, et al Mechanism of all-trans-retinal toxicity with implications for stargardt disease and age-related macular degeneration. J Biol Chem. 2012;287(7):5059–69. 10.1074/jbc.M111.315432 22184108 PMC3281612

[35] Cerignoli F, Guo XJ, Cardinali B, Rinaldi C, Casaletto J, Frati L, et al retSDR1, a short-chain retinol dehydrogenase/reductase, is retinoic acid-inducible and frequently deleted in human neuroblastoma cell lines. Cancer Res. 2002;62(4):1196–204. 11861404

[36] Kam RKT, Shi W, Chan SO, Chen Y, Xu G, Lau CB-S, et al Dhrs3 protein attenuates retinoic acid signaling and is required for early embryonic patterning. J Biol Chem. 2013;288(44):31477–87. 10.1074/jbc.M113.514984 24045938 PMC3814744

[37] Han BC, Xia HF, Sun J, Yang Y, Peng JP. Retinoic acid-metabolizing enzyme cytochrome P450 26a1 (Cyp26a1) is essential for implantation: functional study of its role in early pregnancy. J Cell Physiol. 2010;223(2):471–9. 10.1002/jcp.22056 20112286

[38] Diaz-Gimeno P, Horcajadas JA, Martinez-Conejero JA, Esteban FJ, Alama P, Pellicer A, et al A genomic diagnostic tool for human endometrial receptivity based on the transcriptomic signature. Fertil Steril. 2011;95(1):50–U611. 10.1016/j.fertnstert.2010.04.063 20619403

[39] Lee J, Oh JS, Cho C. Impaired expansion of trophoblast spheroids cocultured with endometrial cells overexpressing cellular retinoic acid-binding protein 2. Fertil Steril. 2011;95(8):2599–601. 10.1016/j.fertnstert.2011.04.065 21605859

