# Supplementary figures and images for: Reprogramming of the retinoic acid pathway in decidualizing human endometrial stromal cells
Source: PLoS One. 2017 Mar 2;12(3):e0173035. doi: 10.1371/journal.pone.0173035 (PMC5333850; doi:10.1371/journal.pone.0173035)

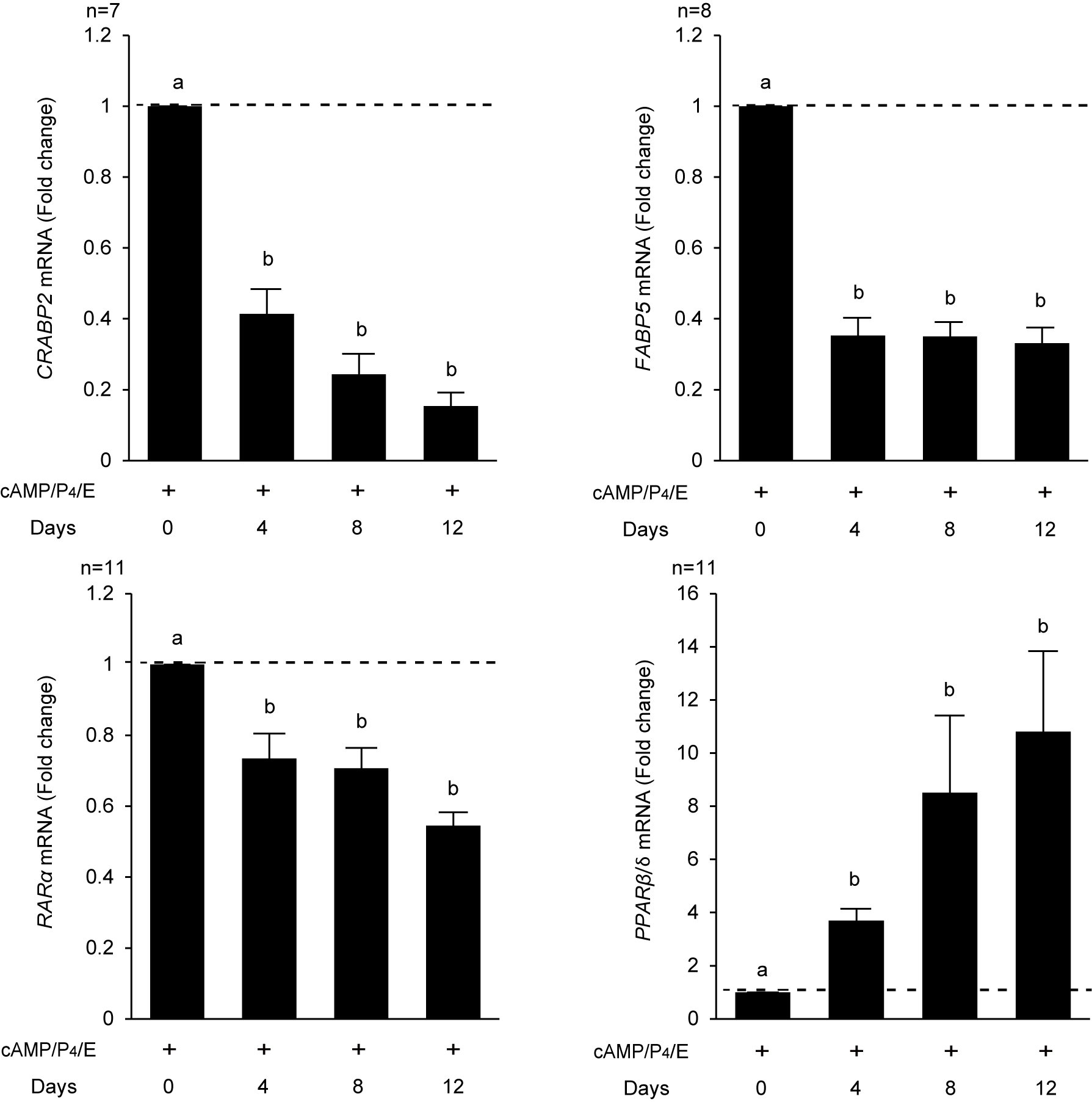

Supplement: S1 Fig — RTQ-PCR analysis of CRABP2, FABP5, RARa and PPAR β/δ transcript levels in decidualizing HESCs for 4, 8 and 12 days. The results show fold-change (mean ± SEM) relative to transcript levels in vehicle-treated control cells (dotted lines). Different letters above the error bars indicate that those groups are significantly different from each other at P < 0.05. The results show mean ± SEM of 7–11 different primary cultures. (TIF) [file pone.0173035.s001.tif]

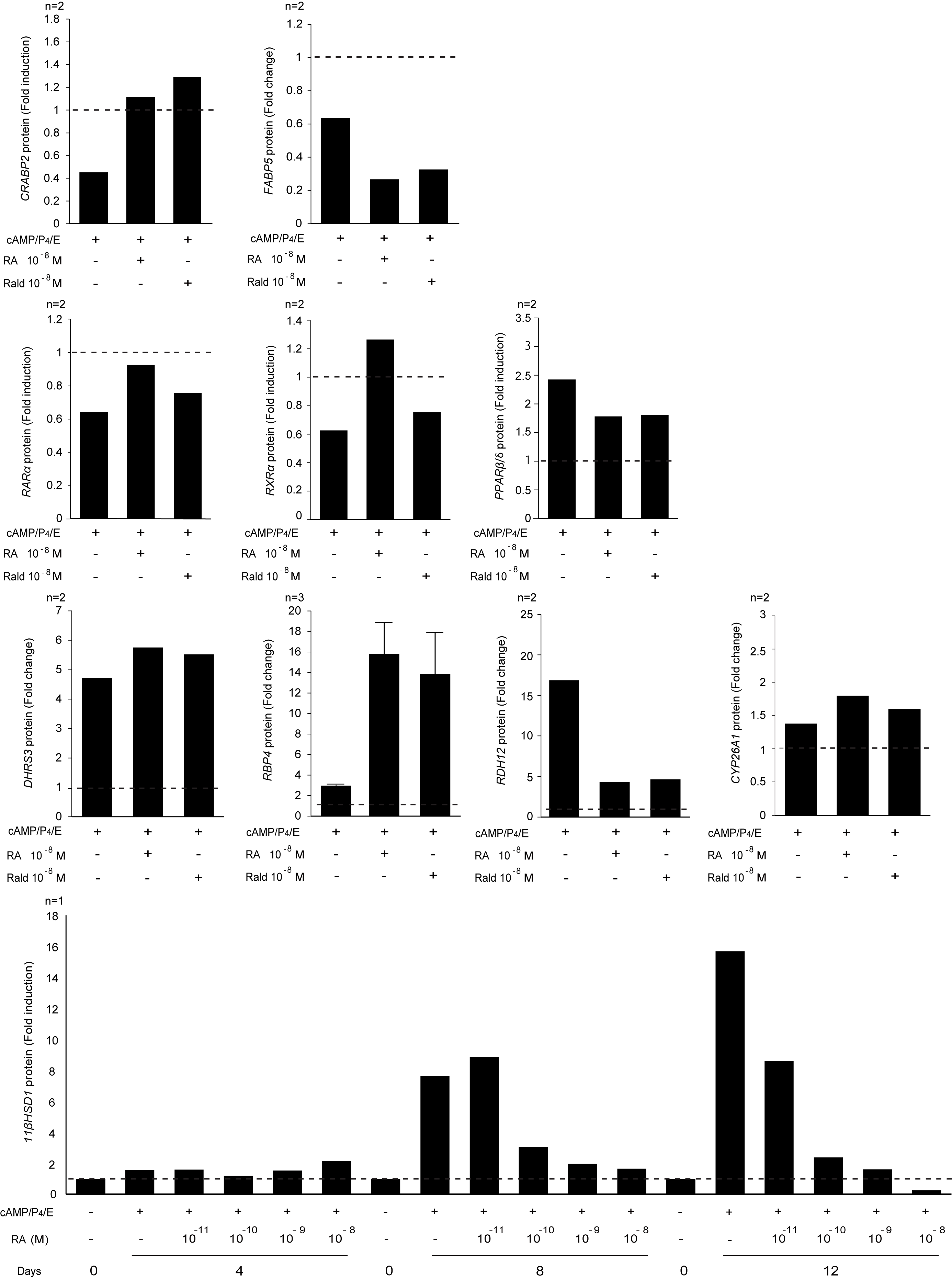

Supplement: S2 Fig — Western blots were quantified by densitometry using ImageJ software. The abundance of the indicated proteins was normalized to β-actin. The data show relative change in protein expression upon decidualization in the presence or absence atRA or Rald exposure was determined. The number of biological repeat experiments are indicated. (TIF) [file pone.0173035.s002.tif]
